# Glioblastoma Formation from Cell Population Depleted of Prominin1-Expressing Cells

**DOI:** 10.1371/journal.pone.0006869

**Published:** 2009-08-31

**Authors:** Kenji Nishide, Yuka Nakatani, Hiroshi Kiyonari, Toru Kondo

**Affiliations:** 1 Laboratory for Cell Lineage Modulation, Center for Developmental Biology, RIKEN, Kobe, Japan; 2 Laboratory for Animal Resources and Genetic Engineering, Center for Developmental Biology, RIKEN, Kobe, Japan; 3 Department of Biology, Graduate School of Science, Kobe University, Kobe, Japan; Cedars-Sinai Medical Center and University of California Los Angeles, United States of America

## Abstract

Prominin1 (Prom1, also known as CD133 in human) has been widely used as a marker for cancer stem cells (CSCs), which self-renew and are tumorigenic, in malignant tumors including glioblastoma multiforme (GBM). However, there is other evidence showing that Prom1-negative cancer cells also form tumors *in vivo*. Thus it remains controversial whether Prom1 is a *bona fide* marker for CSCs. To verify if *Prom1*-expressing cells are essential for tumorigenesis, we established a mouse line, whose *Prom1*-expressing cells can be eliminated conditionally by a Cre-inducible *DTA* gene on the *Prom1* locus together with a tamoxifen-inducible CreER^TM^, and generated glioma-initiating cells (GICs-LD) by overexpressing both the SV40 Large T antigen and an oncogenic H-Ras^L61^ in neural stem cells of the mouse line. We show here that the tamoxifen-treated GICs-LD (GICs-DTA) form tumor-spheres in culture and transplantable GBM *in vivo*. Thus, our studies demonstrate that *Prom1*-expressing cells are dispensable for gliomagenesis in this mouse model.

## Introduction

Recent findings have demonstrated that malignant tumors, including glioblastoma multiforme (GBM), contain cancer stem cells (CSCs), which self-renew and are tumorigenic [1]. Prom1 has been utilized extensively to identify and enrich CSCs from many tumors, including lung cancers, colon cancers, hepatocellular carcinomas, and brain tumors [2]–[6], using the specific anti-Prom1 antibody that recognizes the glycosylated-form of Prom1 [7]. CSCs as well as tissue-specific stem cells (TSCs) in hypoxic niches are likely dormant, and resistant to anti-cancer drugs and irradiation [8], [9]. Moreover, it was shown that TSCs are fostered in these niches [10], [11] and can transform into CSCs when they acquire oncogenic mutations [12]. These, together with the finding that hypoxia induces *Prom1* expression [13], suggest that both TSCs and CSCs would be positive for Prom1 in the niche. However, it remains controversial whether Prom1 is a *bona fide* marker for CSCs as it has been indicated that Prom1-negative glioma cell lines in normoxia become positive for Prom1 in hypoxia, which is one of the characteristics of GBM, and that its expression is reversible upon re-oxygenation [13]. Moreover, there is increasing evidence that Prom1-negative cancer cells from GBMs [14], colon cancers [15], and the Daoy medulloblastoma cell line [16] can form tumors when transplanted *in vivo*. Thus, these findings raise the possibility that CSCs can alter their *Prom1* expression, depending on the culture condition and microenvironment *in vivo*.

In order to confirm whether *Prom1*-expressing cells are essential for tumorigenesis, we established mouse glioma-initiating cell (GIC) lines by overexpressing both SV40 Large T antigen (SV40LT) and a constitutive-active form of H-Ras (HRas^L61^) in neural stem cells (NSCs), whose *Prom1*-expressing cells can be eliminated genetically. We show here that *Prom1* expression is induced in the peripheral cells of tumor-spheres in culture and a portion of glioma *in vivo* as shown in human GBM [5]–[6], [14]. We demonstrate that the induced GIC population depleted of *Prom1*-expressing cells form tumor-spheres in culture and transplantable GBM *in vivo*. Thus, these results suggest that *Prom1*-expressing glioma cells are not essential for tumorigenesis in this mouse model.

## Results and Discussion

### Generation of *Prom1* knock-in mice

**Figure 1:**
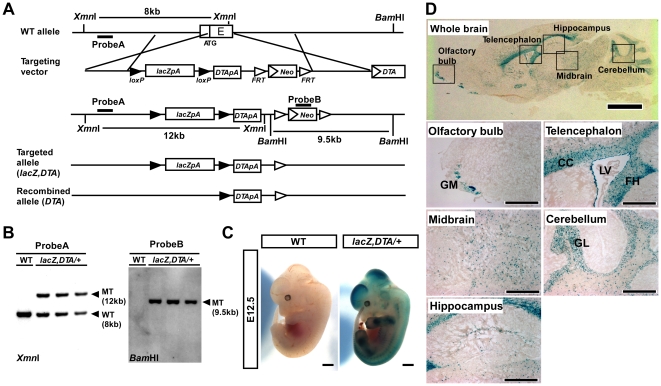
Generation of *Prom1* knock-in mice and reporter analysis in the central nervous system. (A) Schematic representation of targeted allele. (B) Southern blot analysis of three independent heterozygous lines. (C) Whole mount X-gal staining of E12.5 *Prom1^lacZ,DTA/+^* and WT embryos. Scale, 2 mm. (D) X-gal staining of frozen sections of adult mouse brains. Upper panel shows the whole brain and lower five panels display high magnification images of the squared regions in the upper panel. GM, glomeruli; CC, corpus callosum; LV, lateral ventricle; FH, fimbra of hippocampus; GL, granular layer. Scale, 2 mm (upper panel) and 500 µm (lower panels).

We replaced the second exon, which contains the first ATG codon on the *Prom1* locus, with a *floxed-lacZ* and *DTA* (*Prom1^lacZ,DTA/+^*) [17], [18] (Figure 1A and 1B) and generated *Prom1^lacZ,DTA/+^* mice that develop normally and are fertile. We also obtained adult *Prom1^lacZ,DTA/lacZ,DTA^* mice as reported by another *Prom1* knock-in allele [12] and found that they are also viable and develop normally, indicating that Prom1 is not essential for development and our transgene is not toxic.

### *Prom1* expression in embryonic and adult central nervous system

Whole-mount X-gal staining revealed widespread β-galactosidase (β-gal) activity in the developing central nervous system where NSCs exist in abundance (Figure 1C). We detected β-gal activity in the ventricular zone (VZ) of E9.5 embryos to neonatal mice, confirming that *Prom1* is expressed in NSCs in the developing brains (Figure S1). Although immunoreactivity of Prom1 antibody was detected only in the VZ in the adult brain (Figure S2) [19], we found β-gal activity in the cerebellum, midbrain, olfactory bulb, hippocampus, and telencephalon as well (Figure 1D and S2). One possible explanation for the difference observed between our findings and previous findings is that the commercially available anti-Prom1 antibodies recognize limited numbers of Prom1 splicing variants, such as Prom1-s1 [20], whereas the expression of all of the splice variants may be sensitively detected in our knock-in mice.

**Figure 2:**
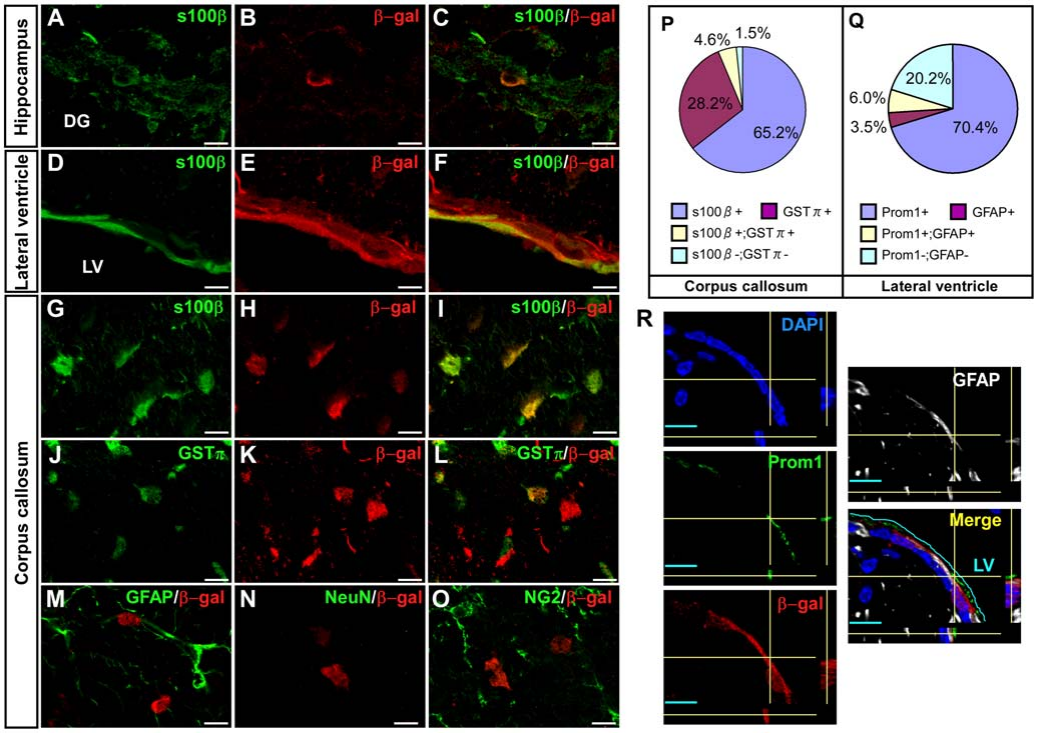
Prom1 is expressed in mature glial cells and SVZ astrocytes in the adult mouse brain. (A–O) Brain sections of adult *Prom1^lacZ,DT-A/+^* were double-labeled for β-gal, which represents *Prom1* expression, and differentiation markers. In hippocampus and ventricular zone, most β-gal positive cells stained for S100β (astrocyte and ependymal cell marker; A–F). In corpus callosum, some β-gal-positive cells labeled for S100β (G–I) and GSTπ (mature oligodendrocytes; J–L) but not for GFAP (another marker for astrocytes; M), NeuN (mature neurons; N), or NG2 (oligodendrocyte precursor cells; O). Scale bars, 10 µm. (P) Quantitative data of differentiation marker-positive cells in β-gal-expressing cells in corpus callosum. Similar trends were observed in the midbrain, neocortex, and other white matter. (Q) Quantitative data of Prom1- and GFAP-positive cells in β-gal-expressing cells in lateral ventricle. (R) In ventricular zone, β-gal-expressing cells (red) were partially labeled for Prom1 (green) and GFAP (white). All nuclei were counterstained with DAPI (blue). Scale bars, 10 µm.

Detailed analyses revealed that Prom1 is expressed in the granular layer of the cerebellum, where the interneurons exist, and in the glomeruli of the olfactory bulb, which are composed of axons of olfactory cells and dendrites of olfactory bulb interneurons. (Figure 1D). We also found that 69% of β-gal-positive cells are labeled for S100β in the hippocampus where NSCs exist in the dentate gyrus (Figure 1D and 2A–2C). In VZ of lateral ventricle, 90% and 9.5% of β-gal-positive cells are labeled for S100β (ependymal cells, Figure 2D–2F) and GFAP (SVZ astrocytes, Figure 2Q), respectively. We also found that 7.9% of GFAP-positive SVZ astrocytes are positive for Prom1 (Figure 2Q and 2R), consistent with the past finding that Prom1 is expressed in multipotent SVZ astrocytes in the adult brain [21]. In the corpus callosum, the β-gal activity was detected in S100β-positive astrocytes and GSTπ-positive oligodendrocytes, but not in NeuN-positive neurons, NG2-positive oligodendrocyte precursor cells, or GFAP-positive astrocytes (Figure 2G–2P). We found similar tendencies in other white matter, neocortex and midbrain as shown in Figure 2P. Thus, these data revealed that Prom1 is expressed in various types of differentiated neural cells as well as NSCs in the adult brain.

### Genetic ablation of *Prom1*-expressing cells by inducible Cre recombinase *in vivo*

**Figure 3:**
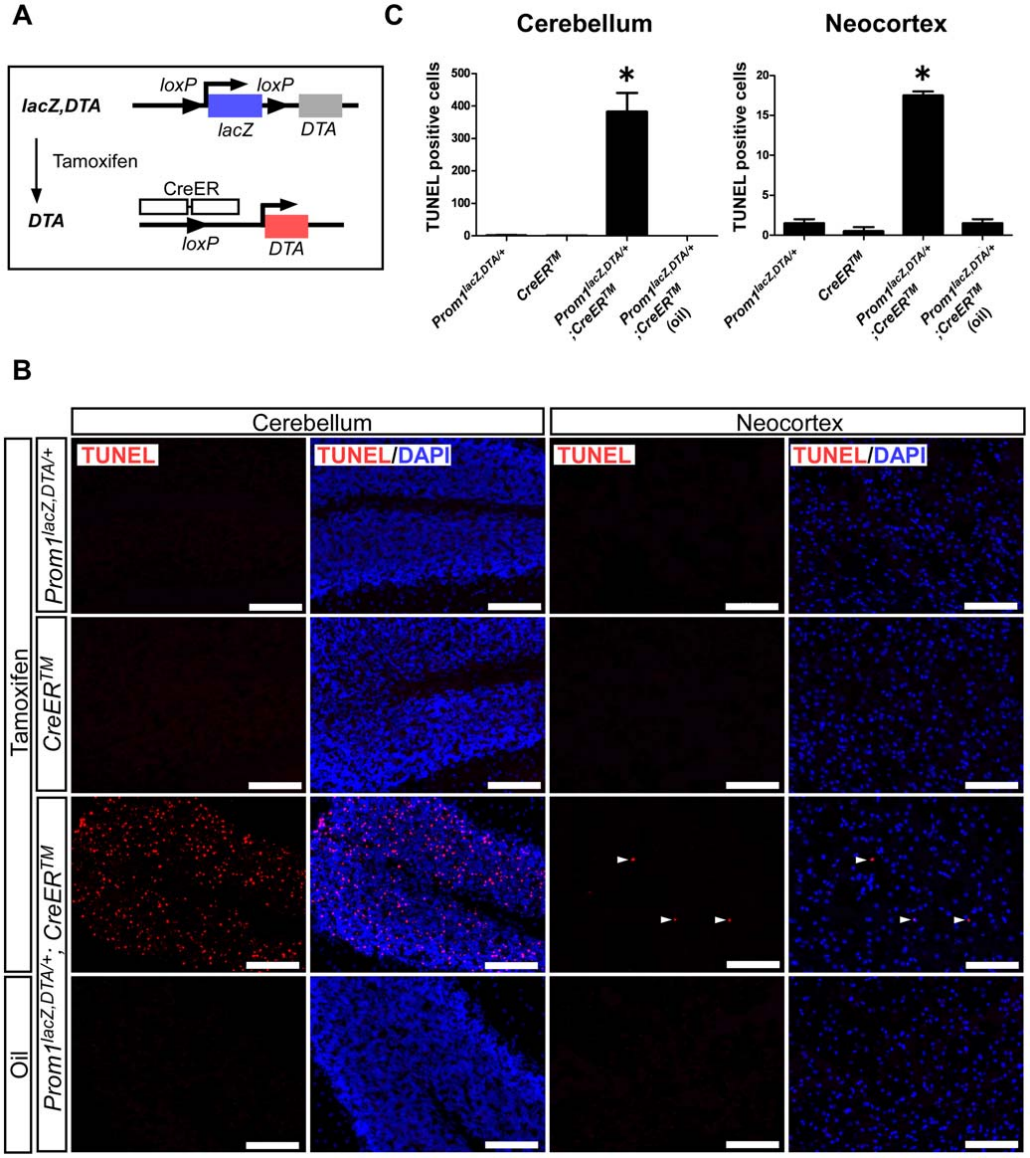
Tamoxifen-dependent Cre activation induces apoptosis of *Prom1*-expressing cells *in vivo*. (A) Schematic diagram of DTA activation. Tamoxifen-dependent Cre activation deleted the *lacZ* gene cassette, leading to the induction of DTA expression upon activation of the *Prom1* promoter. (B) Intraperitoneal injection of tamoxifen-induced cell death (red) in cerebellum (left panels) and neocortex in telencephalon (right panels) as well as midbrain and white matter (not shown). Arrow heads indicate TUNEL-positive cells in the neocortex. All nuclei were counterstained with DAPI (blue). Scales, 100 µm. (C) The number of TUNEL-positive cells per section were counted in the whole neocortex and in half of one lobule of the cerebellum. The results shown are the mean±s.e.m. of two independent experiments. *P<0.05. Similar results were also observed in the midbrain and white matter (not shown).

To eliminate *Prom1*-expressing cells conditionally, the *Prom1^lacZ,DTA/+^* mice were then crossed with the *CAGG-CreER^TM^* transgenic mice that ubiquitously express a tamoxifen-inducible Cre recombinase [22] and generated *Prom1^lacZ,DTA/+^*;*CreER^TM^* double heterozygotes. Upon the activation of Cre recombinase, the *floxed-lacZ* cassette is cut off, leading to the specific elimination of the *Prom1*-expressing cells by DTA induced upon the activation of *Prom1* promoter (Figure 3A). We first examined whether this experimental system works *in vivo*. As expected, after five consecutive days of intraperitoneal injections of tamoxifen, we induced a number of TUNEL-positive cells in the granular layer of cerebellum, cortex of telencephalon, white matter, and midbrain (Figure 3B, 3C and not shown). In addition, tamoxifen-injected mice lost body weight and showed a walking abnormality caused by functional defects of the cerebellum. They did, however, survive at least 15 days after the final injection (Figure S3A). It will be of interest to further investigate how the elimination of *Prom1*-expressing cells causes body weight loss in mice. Nonetheless, we could not eliminate *Prom1*-expressing cells in VZ of the mice and detected migrating neuroblasts in the rostral migratory stream (Figure S3B, arrow) and olfactory bulb, suggesting that neurogenesis was taking place in the mice (Figure S3B). These data indicate that our experimental system works as expected, although the efficiency of DTA induction is dependent on the region or cell types.

### Establishment of a mouse model for GBM

**Figure 4:**
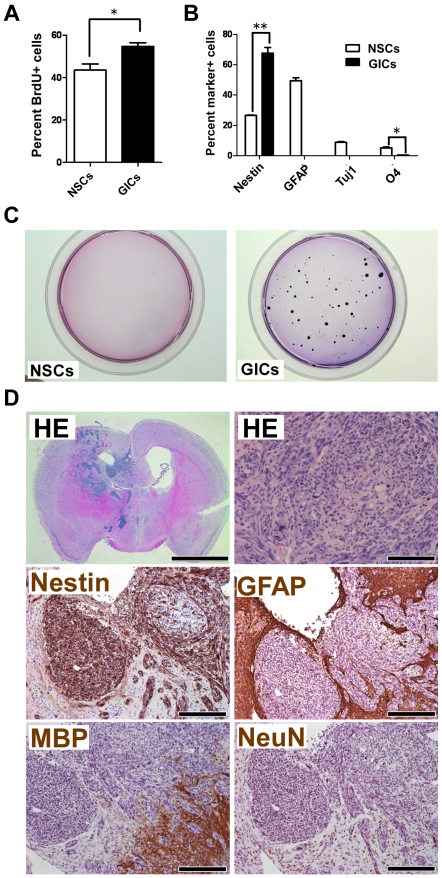
Characteristics of the GICs-LD. (A) Proliferation of the GICs-LD and their parental cells (NSCs) was determined by BrdU incorporation assay. The results shown are the mean±SD of three cultures. *P<0.05. (B) The proportion of neural lineage marker-positive cells is shown as the mean±SD of two cultures. *P<0.05; **P<0.01. (C) Colony formation ability was examined in soft agar. (D) Brain sections with tumors derived from GICs-LD were stained with hematoxylin and eosin (HE) and for Nestin, GFAP, MBP, and NeuN. Scales, 2 mm (upper left panel), 100 µm (upper right panel), and 200 µm (middle and lower panels).

To verify whether *Prom1*-expressing cells are essential for tumorigenesis, we adopted a GIC transplantation method into nude mice rather than tumor induction in the *Prom1^lacZ,DTA/+^*;*CreER^TM^* mice because the DTA induction efficiency is low in the brain as mentioned above (Figure S3B). To generate a mouse GIC line, we first transfected NSCs from *Prom1^lacZ,DTA/+^*;*CreER^TM^* line with pBabe-Puro-SV40LT vector, which blocks both p53 and Rb pathways, as high frequencies of mutations in p53 (87%) and Rb (78%) pathways are seen in human GBM [23], [24]. Then the SV40LT-expressing NSCs were transfected with pCMS-EGFP-H-Ras^L61^ vector, as an increased activation of the Ras signaling pathway is detected in about 90% of human GBM [25], to establish GIC-*Prom1^lacZ,DTA/+^*;*CreER^TM^* line (GIC-LD). GICs-LD proliferated faster than their parental NSCs as confirmed by the BrdU incorporation assay (Figure 4A). We also found that when both types of cells were cultured under differentiation conditions, GICs-LD did not show any signs of undergoing differentiation, while their parental cells were labeled for the neuronal marker Tuj1, glial markers O4 and glial fibrillary acidic protein (GFAP), and NSC markers Nestin and Sox2 (not shown) (Figure 4B). Moreover, GICs-LD formed colonies in soft agar whereas their parental cells did not (Figure 4C). Together, these data suggest that GICs-LD are transformed and do not readily differentiate *in vitro*.

We then addressed whether GICs-LD form tumors *in vivo*. We injected 10^4^ cells into brains of nude mice and found that they formed tumors with histological features similar to human GBM, including hypercellularity, pleomorphism, multinuclear giant cells, mitosis, and necrosis [26] (Figure 4D). In addition, we noticed that the tumors were comprised of Nestin-positive cell populations, rather than a mixture of NSC marker- and differentiation marker-positive cells, GFAP+ astrocytes, myelin basic protein (MBP)+ mature oligodendrocytes, and NeuN+ mature neurons, indicating that the tumor cells maintain characteristics of NSCs and are unlikely to differentiate *in vivo* (Figure 4D). We also confirmed these results by immunolabeling the tumors for GFP and neural markers (Figure S4).

**Figure 5:**
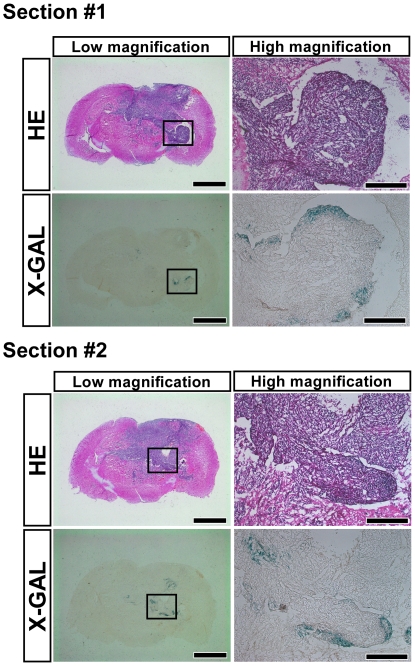
Prom1 is expressed in the periphery of brain tumors. Serial brain sections with tumors were stained with HE (upper panels) and X-gal (lower panels). Section #1 and #2 were from two independent regions of the brain. Right panels indicate the high magnification images of the squared region of left panels. X-gal-positive cells were detected in the peripheral region of tumors. Scales, 2 mm (left panels) and 200 µm (right panels).

We found *Prom1*-expression in a portion of brain tumors, especially in the peripheral region of the tumors (Figure 5), consistent with the previous findings that human GBM contain a small population of Prom1-positive cells [5]–[6], [14]. To determine whether *Prom1*-expressing cells reside in the hypoxic region, we immunolabeled different sections with either CD31 for blood vessels or HIF1α for hypoxia in combination with X-GAL staining. Although *Prom1*-expressing cells were not labeled for HIF1α, they were adjacent to the HIF1α-positive hypoxic regions (Figure S5). In contrast, *Prom1*-expressing cells were not detected around or near blood vessels (Figure S5). Thus, these data suggest that *Prom1* expression may be regulated by a HIF1α-independent hypoxic signal pathway.

### The GIC population lacking *Prom1*-expressing cells can form tumor-spheres *in vitro*

**Figure 6:**
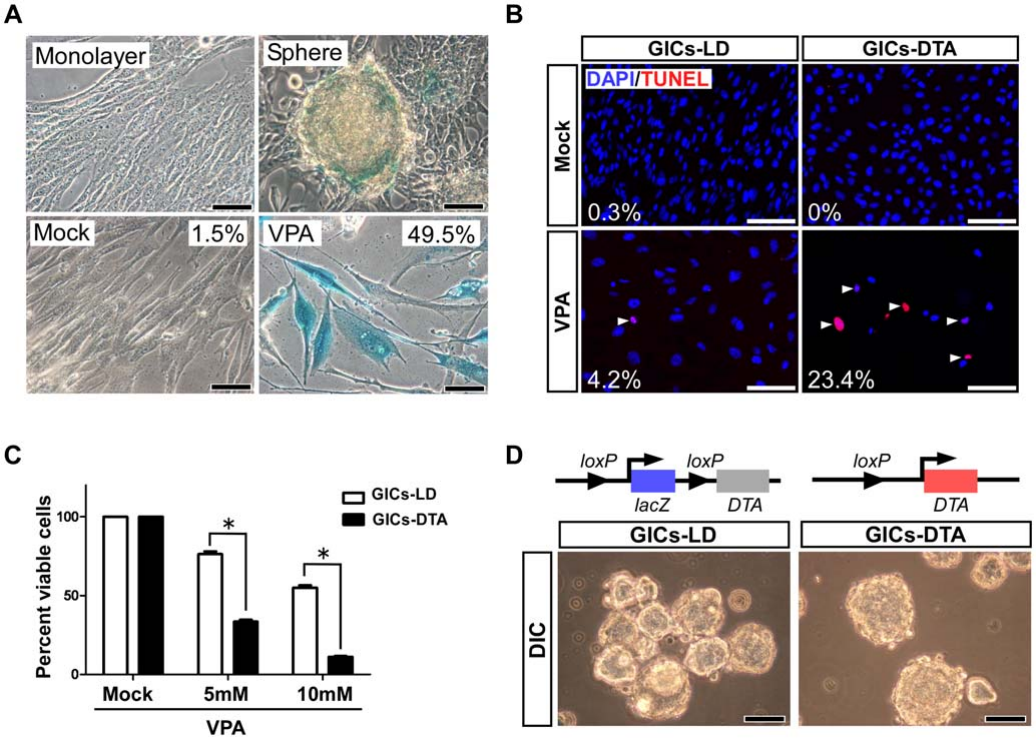
*Prom1*-expressing cells are dispensable for maintaining GICs in culture. (A) X-gal staining of monolayer-cultured GICs-LD (Monolayer), their tumor spheres (Sphere), and valproic acid (VPA, 10 mM)-treated cells (lower panels; representative data from three cultures). (B) VPA induced TUNEL-positive cell death in GICs-DTA. All nuclei were counterstained with DAPI (blue). Scales, 100 µm (C) Survival of GICs-LD and GICs-DTA was determined by MTT assay. The results shown are the mean±s.e.m of three cultures. *P<0.001. (D) Sphere formation of GICs-LD and GICs-DTA. Upper schemes indicate the structures of the genome. Scale, 60 µm.

We also found that very few GICs-LD were LacZ-positive when cultured as monolayers, whereas a significant number of these cells expressed LacZ when they formed spheres, suggesting that the Prom1 promoter is activated by cell adhesion and cell-to-cell communication (Figure 6A). To test whether the *Prom1*-expressing cancer cells are essential for tumorigenesis in this system, we generated GICs-*Prom1^DTA/+^*;*CreER^TM^* (GICs-DTA) by culturing GICs-LD as a monolayer with 4-hydroxy-tamoxifen, and then established two independent GICs-DTA sublines using limiting dilution methods. The genotype of the sublines was confirmed with genomic PCR analysis (not shown). As shown by Tabu et al [27], we confirmed that when the *Prom1* promoter was activated by a histone deacetylase inhibitor, valproic acid (VPA, 10 mM), over 90% of the cells in GICs-DTA sublines died within 2 days, while over 60% of their parental GICs-LD lines survived under the same conditions (Figure 6A–6C). It was believed that Prom1-positive cells are required for the formation of tumor-spheres *in vitro* [8]–[9], [14], however, we found that GICs-DTA sublines can proliferate and form spheres with no observable defects (Figure 6D), indicating that *Prom1*-expressing cells are not essential for the maintenance of GIC population in culture.

### GBM formation from the cell population depleted of *Prom1*-expressing cells

**Figure 7:**
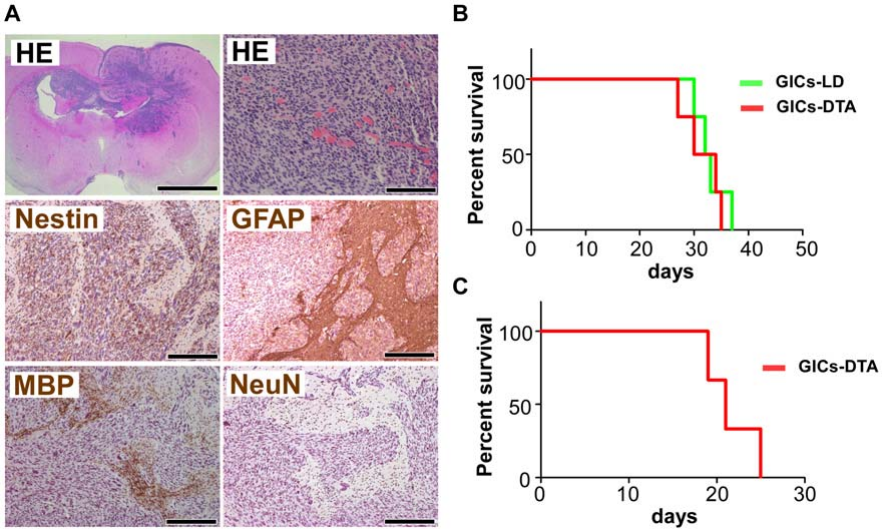
*Prom1*-expressing cells are not required for the tumorigenesis of GICs in nude mice. (A) Brain sections with tumors derived from GICs-DTA. The GICs invaded into the brain parenchyma. HE staining of the tumors shows necrosis, hypercellularity and hypervascularity, multinuclear giant cells, and mitotic cells; these pathological features are similar to human GBM (upper panels). Immunohistochemical analysis of the tumor for Nestin, GFAP, MBP, and NeuN (middle and lower panels). Scales, 2 mm (upper left panel), 100 µm (upper right panel), and 200 µm (middle and lower panels). (B) Survival curves for mice (n = 4 of each line) injected with 10^4^ GICs-LD (green line) and GICs-DTA (red line). No significance was observed, *P* = 0.65. (C) Survival curve for mice (n = 3) serially transplanted with 10^4^ GFP-positive primary tumor cells.

GICs-DTA sublines were then transplanted into the brains of nude mice. Both sublines produced brain tumors with similar characteristics to their control tumors (Figure 4D and 7A) and human GBM [26], and led to the death all of the mice like their parental GICs-LD (n = 4 for each cell lines, P = 0.65) (Figure 7B). We could not detect LacZ-positive cells in the tumors, confirming that tumors derived from GICs-DTA sublines do not have any *Prom1*-expressing cells (not shown). Furthermore, we performed serial transplantation experiments, and found that all of the mice (n = 3) that received secondary transplantation developed brain tumors that were phenocopies of primary ones (data not shown) and died within 30 days (Figure 7C), indicating that GIC-DTA sublines have high capacity for self-renewal. Taken together, these data reveal that *Prom1*-expressing cancer cells are not essential for tumorigenesis and its maintenance in this GBM model.

Using the *Prom1* knock-in allele, we demonstrated that *Prom1* is predominantly expressed in differentiated cells as well as NSCs in the adult brain and that mouse GIC populations that eliminate *Prom1*-expressing cells can proliferate in culture and form transplantable GBM *in vivo*, suggesting that Prom1 is not a specific marker for NSCs or GICs. However, we cannot exclude the possibility that Prom1-positive glial cells may behave as multipotent NSCs as shown previously [19], [28] and that Prom1-positive GICs may be more malignant than other GICs and are essential for tumorigenesis in other glioma models. It also remains to be evaluated whether human GIC population depleting *Prom1*-expressing cells can form malignant glioma *in vivo*. Using the *Prom1^lacZ,DTA/+^* mice, it is important to clarify whether *Prom1*-expressing cells are essential for the tumorigenesis of other cancers, including breast, intestinal, and prostate cancers. Thus our *Prom1^lacZ,DTA/+^* mice will be a useful tool for the research community to examine the functions of *Prom1*-expressing cells, which exist throughout the adult body, by crossing them with other transgenic mice that express inducible and tissue-specific Cre recombinase.

## Materials and Methods

### Mice and Chemicals

*Prom1* knock-in mice (Acc. No. CDB0623K: http://www.cdb.riken.jp/arg/mutant%20mice20list.html) were generated using a knock-in method as described [29]. To prevent leaky expression of DTA, a double polyA signal was inserted under the *lacZ* gene (http://www.cdb.riken.jp/arg/cassette.html). *Prom1* knock-in mice that were backcrossed and maintained on a C57BL/6 genetic background, were crossed with *ACTB-FLPe* mice [30] to delete the neomycin selection cassette to generate *Prom1^lacZ,DTA/+^* mice. *Prom1^lacZ,DTA/+^* mice were further crossed with *CAGG-CreER^TM^* mice to delete the *lacZ* cassette upon Cre activation, generating *Prom1^lacZ,DTA/+^*;*CreER^TM^* mice. Genotypes of each mouse were confirmed by Southern blot analysis and PCR. To induce CreER activation, tamoxifen (Sigma), which was dissolved in sun-flower oil (20 mg/ml), was injected intraperitoneally into mice (9 mg/40 g body weight) for 5 consecutive days. The mice were dissected and analyzed 10 days after the final injection. All mouse experiments were performed following the protocols approved by the RIKEN CDB Animal Care and Use Committee.

Chemicals and growth factors were purchased from Sigma and PeproTech, respectively, except where indicated.

### Intracranial cell transplantation into the brain of nude mice

Control cells and transformed cells were suspended in 5 µl of culture medium and injected into brains of 5∼8 week-old female nude mice that had been anesthetized with 10% pentobarbital. The stereotactic coordinates of the injection site were 2 mm forward from lambda, 2 mm lateral from the sagittal suture, and 5 mm deep.

For serial transplantation, brain tumors were dissociated enzymatically using the papain dissociation system (Worthington). GFP-positive transformed cells were purified using a JSAN flow cytometer (BayBioscience, Japan), suspended in culture medium, and transplanted into nude mice soon after sorting.

### Cell culture

NSCs were prepared from embryonic day 17.5 *Prom1^lacZ,DTA/+^*;*CreER^TM^* mouse telencephalon and cultured in DMEM/F12 (Gibco, BRL) supplemented with chemicals, bFGF (10 ng/ml), and EGF (10 ng/ml) (NSC medium), as described previously [31]. For the monolayer culture, cells were cultured on poly-D-lysine- (PDL, 15 µg/ml) and fibronectin- (1 µg/ml, Invitrogen) coated dishes in NSC medium or on non-coated dishes in NSCs medium with 5% FCS.

To induce neural differentiation, NSCs were cultured on 8-well chamber slides (Nunc) in DMEM with 1% FCS for one week. *Prom1* expression was induced in the cells cultured with 10 mM Valproic acid (VPA) (Calbiochem) for 3 days. BrdU incorporation assay was performed as described previously [32]. To isolate GICs-DTA sublines, GICs-LD were cultured with 4-hydroxy-tamoxifen (100 nM) for 4 days. The single cells were then isolated and cultured in the 96-well dishes to avoid contamination of non-recombinant cells. Deletion of the *lacZ* gene in GICs-DTA sublines was confirmed by PCR analysis (see below).

### Southern blot analysis and genotyping

To determine the genome structure of *Prom1* knock-in mice, genomic DNA was digested with *Xmn*I or *Bam*HI. Digoxigenin (DIG)-labeled external or internal probes were generated using PCR DIG Probe Synthesis kit (Roche). Detailed probe sequences will be disclosed on request. Hybridization was conducted using standard protocols. In addition to the external probes (probe A), internal probes (probe B) were used to detect random integrations of the targeting vector on the genome.

Genotyping of the knock-in mice was done by PCR. Genomic DNA was isolated from the mouse tail by standard methods and then used for PCR. The following oligonucleotide DNA primers were synthesized: For *Prom1* knock-in allele, prom1-fwd: 5′-GCT TGAGAGATCAGG CCAACAAC-3′, prom1-rvs: 5′-CTAGAGGGAAGTCATTCGGCTG-3′ and lacZas: 5′-CGCGTAAAAATGCGCTCAGG-3′. The primers for *CAGG-CreER^TM^* and *ACTB-FLPe* were as described previously [22], [30]. Dimethylsulfoxide (DMSO, 5%) was added to the all reaction mixture. Cycle parameters were 20 sec at 94°C, 30 sec at 60°C, and 40 sec at 72°C for 35 cycles.

RT-PCR was carried out as described previously [32]. For *gapdh*, cycle parameters were 10 sec at 94°C, 20 sec at 58°C, and 40 sec at 72°C for 25 cycles. DNA primers for *Prom1* were synthesized: the 5′ primer was 5′-AGGCTACTTTGAACATTATCTGCA-3′ and the 3′ primer was 5′-GGCTTGTCATAACAGGATTGT-3′. Cycle parameters were 10 sec at 94°C, 20 sec at 58°C, and 40 sec at 72°C for 40 cycles.

### Immunochemistry

Immunostaining was carried out as described previously [32], [33]. The following antibodies were used to detect antigens: rat anti-Prom1 (1∶100; eBioscience), mouse anti-BrdU (1∶1000; Hybridoma Bank), rabbit anti-GFAP (1∶500; DakoCytomation), mouse anti-Nestin (1∶500; BD Pharmingen), mouse anti-beta tubulin isotype III (Tuj1; 1∶200; Sigma), mouse anti-O4 (1∶5; hybridoma supernatant; ATCC), mouse anti-MBP (1∶1000; COVANCE), mouse anti-NeuN (1∶200; Chemicon), rabbit anti-GFP (1∶500; Abcam), rat anti-GFP (1∶500; Nacalai Tesque, Japan), chicken anti-β-gal (1∶500; Abcam), rabbit anti-NG2 (1∶200; Chemicon), rabbit anti-S100β (1∶500; DakoCytomation), mouse anti-GSTπ (1∶100; BD Pharmingen), mouse anti-PSA-NCAM (1∶500; gift from T. Seki, Juntendo University School of Medicine), mouse anti-HIF1α (1∶500; Abcam) and rabbit anti-CD31 (1∶50; Abcam). The antibodies were detected with goat anti-rabbit IgG-A488, goat anti-mouse IgG-A488 (1∶400; Invitrogen), goat anti-mouse IgG-Cy3, donkey anti-rabbit IgG-Cy3, donkey anti-mouse IgG-Cy3, donkey anti-chicken IgY-Cy3, donkey anti-rabbit IgG-Cy5 (1∶200; Jackson ImmunoResearch), and rabbit anti-mouse IgM-Texas-Red (1∶200; Jackson ImmunoResearch). To visualize the nuclei, the cells were counterstained with DAPI (1 µg/ml).

### Soft agar assay

We performed a soft agar assay to examine whether the cultured cells could proliferate anchorage-independently. The cells were suspended in 0.3% top agar containing optimum medium and layered onto 0.6% bottom agar made with the same medium. After the top agar solidified, culture medium was added and the cells were cultured for 20 days with medium changes every 3 days.

### MTT assay

One thousand cells were cultured in 100 µl of culture medium in each well of the 96-well plates with or without VPA (5 or10 mM) for 2 days. The cells were cultured in the presence of MTT labeling reagent (Roche) for 4 h and then incubated overnight at 37°C with the solubilization solution (10% SDS in 0.01 M HCl). The viable cells were quantified on a microplate reader (Bio-Rad) with the absorption spectrum at 595 nm.

### Brain fixation and histopathology

The dissected mouse brains were fixed in 4% paraformaldehyde overnight at 4°C. After fixation, the brains were cryoprotected with 12–18% sucrose in PBS and embedded in Tissue-Tek OCT compound (Miles, Elkhart, IN). Coronal sections (10 µm thick) were prepared from the cerebral cortex and stained with hematoxylin-eosin (HE) using a standard technique.

For X-gal staining, frozen sections (10 µm thick) were fixed by 0.2% glutaraldehyde in PBS or 4% paraformaldehyde in PBS for 5 min, washed several times, and stained X-gal staining solution (2 mg/ml X-gal) for 24 hours.

Immunohistochemical analysis was carried out using the standard ABC method (VECTOR) and fluorescent-dye conjugated secondary antibodies described above. Primary antibodies used in this experiment are described above. For Nestin immunostaining, antigen was retrieved by HistoVT One according to the supplier's instructions (Nacalai Tesque). After the second antibody treatment, samples were incubated in peroxidase substrate solution and then counterstained with hematoxylin. TUNEL assay was carried out as described previously [33].

### Transfection

Transfection was performed using the Nucleofector system, according to the supplier's instructions (Amaxa). In brief, 2×10^6^ cells were suspended in the Mouse NSC Nucleofector Solution (100 µl) with 10 µg vectors, and were then transfected using the Nucleofector Device. Transfected cells were cultured in their optimized medium and selected with puromycin (0.25 µg/ml) and hygromycin (100 µg/ml). GFP-positive transfected cells were then purified using a flow cytometer (JSAN) as previously described [33].

### Vector construction

Full-length SV40LT cDNA was amplified from pBS-SVT (Japan Health Sciences Foundation, Tokyo, Japan) using PCR and Phusion polymerase (FINNZYME, Espoo, Finland), according to the manufacturer's instructions. Full-length mouse *H-Ras* was amplified from mouse NSC cDNA using RT-PCR and Phusion polymerase, and *H-Ras^L61^* was made by substituting a leucine for the glycine at codon 61 by PCR. Amplified cDNA were cloned into a pDrive vector (QIAGEN). The nucleotide sequences were verified using the BigDye Terminator Kit version 3.1 (Applied Biosystems) and an ABI sequencer model 3130xl (Applied Biosystems). *H-Ras^L61^* and SV40LT cDNA were inserted into pCMS-EGFP vector and pBabe-Puro, resulting in pCMS-EGFP-*H-Ras^L61^* and pBabe-Puro-SV40LT, respectively. The following oligonucleotide DNA primers were synthesized: For the full-length mouse *H-Ras*, the 5′ primer was 5′-TGAATTCGCCACCATGACAGAATACAAGCTTGTGGTG-3′ and the 3′ primer was 5′-ACTCGAGTCAGGACAGCACACATTTGCAG-3′. For *H-Ras^L61^*, the 5′ primer was 5′-ACAGCAGGTCTAGAAGAGTATA-3′ and the 3′ primer was 5′-TATACTCTTCTAGACCTGCTGT-3′. For *SV40LT*, the 5′ primer was 5′-AGAATTCGCCACCATGGATAAAGTTTTAAACAGAGAG-3′ and the 3′ primer was 5′-AGTCGACTTATGTTTCAGGTTCAGGGGG-3′.

### Statistical analysis

Survival probability was calculated and plotted by Kaplan-Meier methods. The difference was analyzed using the logrank test. Comparison of cell growth, differentiation, and X-GAL induction were performed by the unpaired Student's *t*-test. Comparison of cell viability and cell death after VPA treatment was performed two-way ANOVA followed by Bonferroni posttests. All statistic analysis was performed using software GraphPad Prism 5.0 (Graphpad Software).

## Supporting Information

Figure S1 Developmental expression pattern of Prom1. X-gal staining (blue) of frozen sections of E9.5, E12.5, E14.5 and P0 Prom1lacZ,DTA/+ mouse brains. Sections were counterstained with eosin (red) to visualize cytoplasm. Scale, 200 µm.
(4.28 MB TIF)

Figure S2 Prom1 antibody labels Prom1-expressing cells in VZ but not in the other regions. Brain sections of adult Prom1lacZ,DTA/+ mice were immunolabeled for β-gal (red) and Prom1 (green). LV, lateral ventricle; DG, dentate gyrus; GL, granular layer. All nuclei were counterstained with DAPI (blue). Scale, 100 µm.
(2.02 MB TIF)

Figure S3 Tamoxifen injection decreases body weight of Prom1lacZ,DTA/+;CreERTM mice but does not affect VZ cells and their neurogenesis. (A) Relative body weight of mice. Tamoxifen was injected once a day for five consecutive days and body weight were measured every five days up to fifteen days after final injection. PC-TM, tamoxifen-injected Prom1lacZ,DTA/+;CreERTM mouse; PC-oil, oil-injected Prom1lacZ,DTA/+;CreERTM mouse; CreER-TM, tamoxifen-injected CreERTM mouse; Prom1-TM, tamoxifen-injected Prom1lacZ,DTA/+ mouse. (B) Upper panels show X-gal staining of frozen sections of tamoxifen-injected Prom1lacZ,DTA/+;CreERTM mouse brains. Lower four panels indicate PSA-NCAM immunostaining (red) in the ventricular zone and olfactory bulbs in tamoxifen-injected Prom1lacZ,DTA/+;CreERTM mice. Arrows show migrating neuroblasts in rostral migratory stream. All nuclei were counterstained with DAPI (blue). LV, lateral ventricle. Scale, 100 µm.
(2.25 MB TIF)

Figure S4 Brain tumors derived from GICs-LD were labeled for Nestin but not differentiation markers. Brain sections with tumors were immunolabeled for GFP (GICs, green) and either neural stem cell marker (Nestin, red) or differentiation markers, GFAP (astrocytes, red), MBP (oligodendrocytes, red), NeuN (mature neurons, red) and NG2 (oligodendrocytes precursor cells, red). Although most of GFP-positive cells were NG2-negative, very few double-positive cells were detected. Scale, 100 µm.
(5.61 MB TIF)

Figure S5 Prom1-expressing cells were adjacent to hypoxic regions but not to blood vessel. Frozen sections of brain tumors were stained with X-gal activity and then immunoelabeled for GFP (green) and either CD31 (marker for endothelrial cells, red) or HIF1α (marker for hypoxia, red). Arrow heads indicate CD31-positive cells. Scale, 100 µm.
(9.60 MB TIF)
