# Correction: Glioblastoma Formation from Cell Population Depleted of Prominin1-Expressing Cells

**DOI:** 10.1371/annotation/d1e654f7-c02d-458c-8dc7-da7ae96aa594

**Published:** 2009-09-24

**Authors:** Kenji Nishide, Yuka Nakatani, Hiroshi Kiyonari, Toru Kondo

The affiliation of the first author was incorrect. Kenji Nishide should be affiliated not with #2 but with #3 Department of Biology, Graduate School of Science, Kobe University, Kobe, 657-8601, Japan. 

