# Supplementary figures and images for: Glioblastoma Formation from Cell Population Depleted of Prominin1-Expressing Cells
Source: PLoS One. 2009 Aug 31;4(8):e6869. doi: 10.1371/journal.pone.0006869 (PMC2729925; doi:10.1371/journal.pone.0006869)

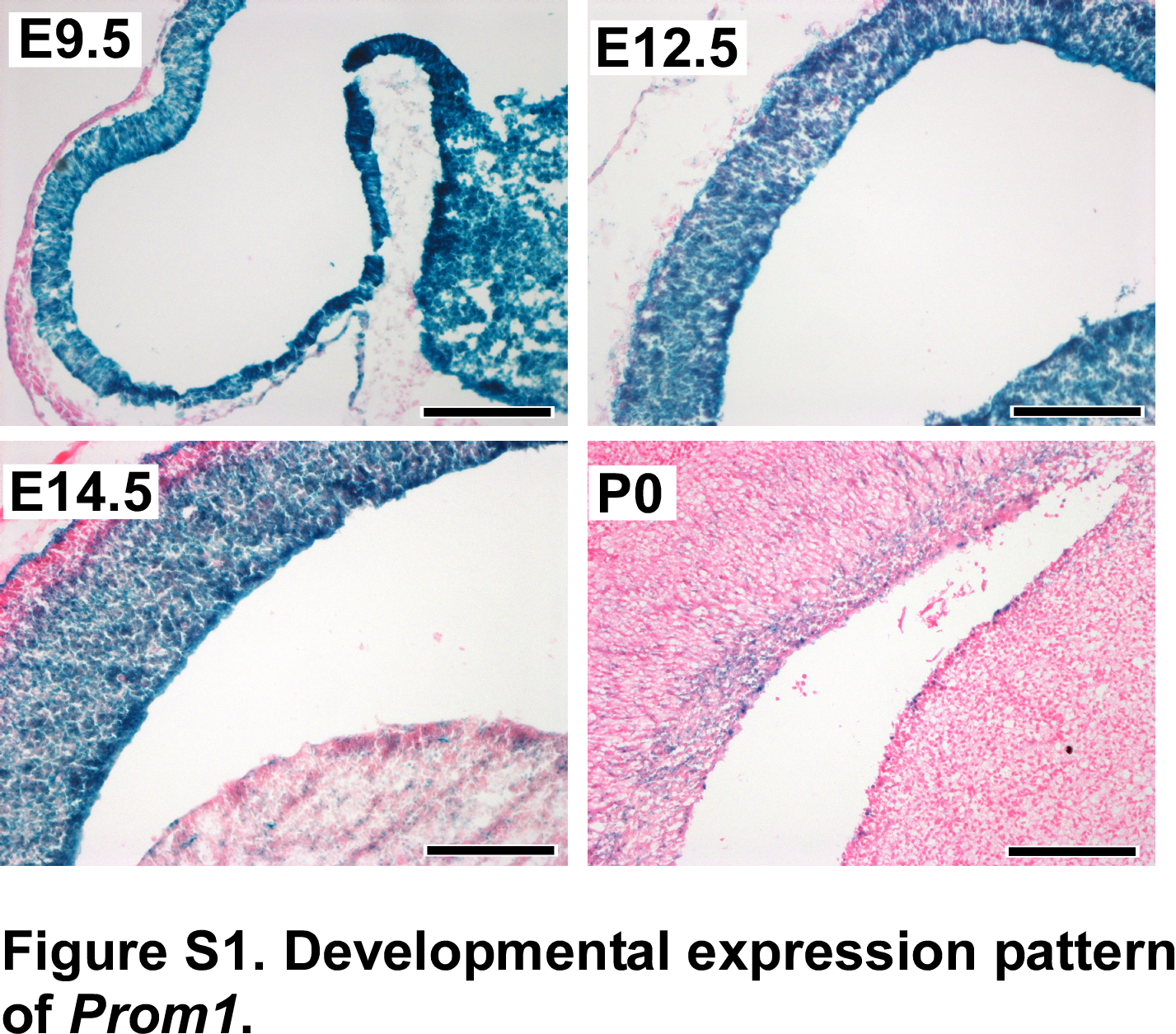

Supplement: Figure S1 — Developmental expression pattern of Prom1. X-gal staining (blue) of frozen sections of E9.5, E12.5, E14.5 and P0 Prom1lacZ,DTA/+ mouse brains. Sections were counterstained with eosin (red) to visualize cytoplasm. Scale, 200 µm. (4.28 MB TIF) [file pone.0006869.s001.tif]

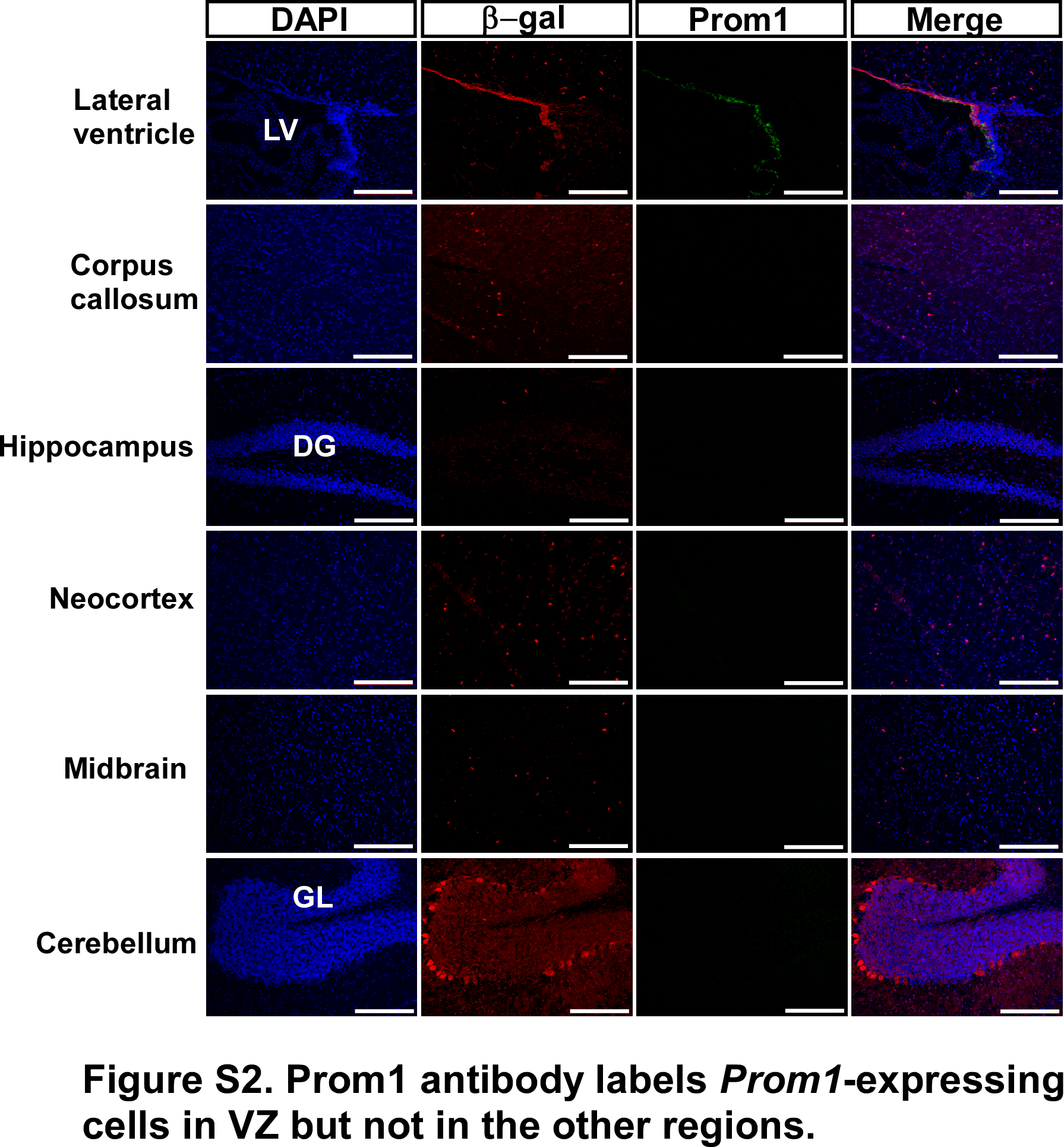

Supplement: Figure S2 — Prom1 antibody labels Prom1-expressing cells in VZ but not in the other regions. Brain sections of adult Prom1lacZ,DTA/+ mice were immunolabeled for β-gal (red) and Prom1 (green). LV, lateral ventricle; DG, dentate gyrus; GL, granular layer. All nuclei were counterstained with DAPI (blue). Scale, 100 µm. (2.02 MB TIF) [file pone.0006869.s002.tif]

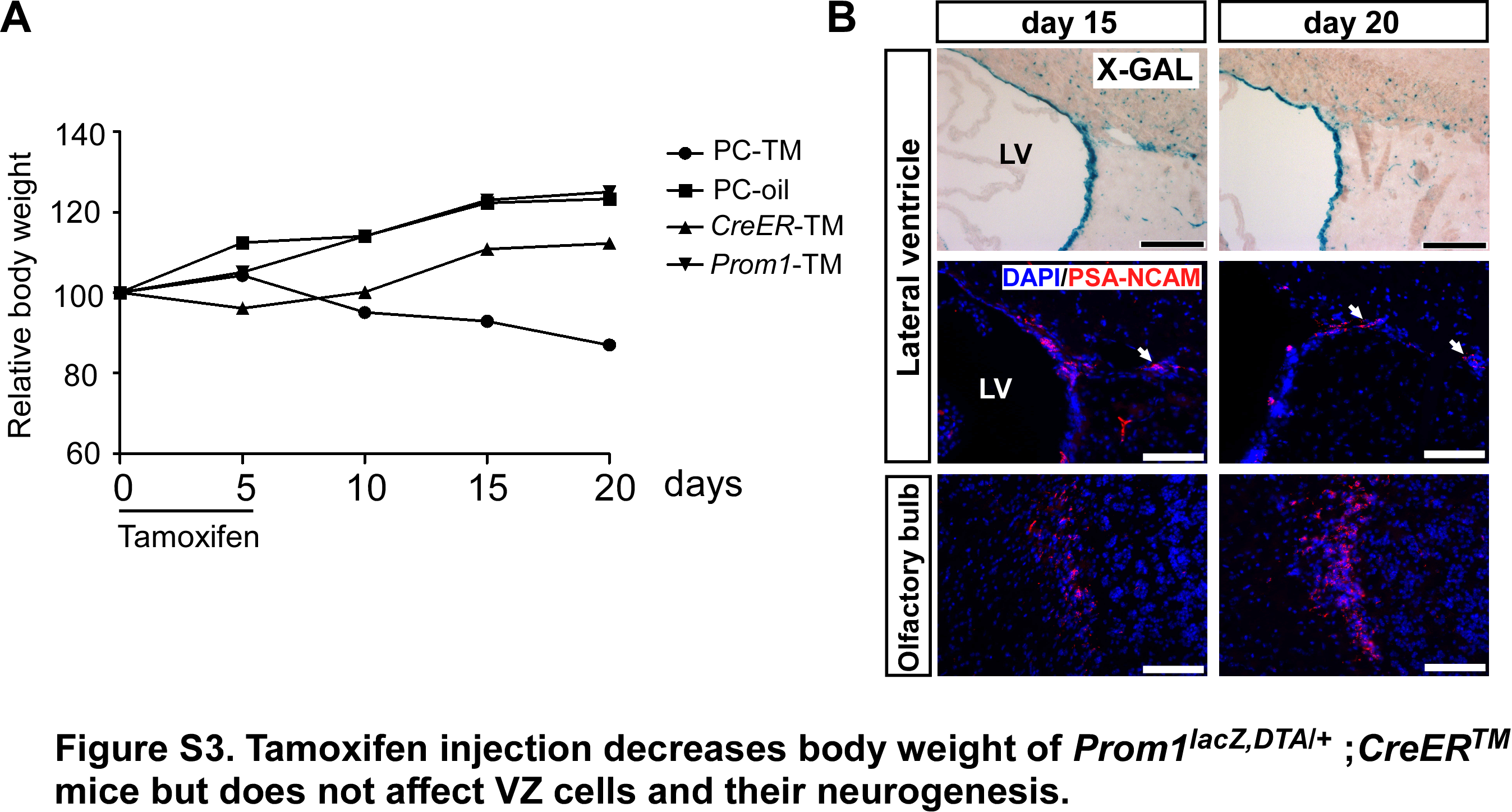

Supplement: Figure S3 — Tamoxifen injection decreases body weight of Prom1lacZ,DTA/+;CreERTM mice but does not affect VZ cells and their neurogenesis. (A) Relative body weight of mice. Tamoxifen was injected once a day for five consecutive days and body weight were measured every five days up to fifteen days after final injection. PC-TM, tamoxifen-injected Prom1lacZ,DTA/+;CreERTM mouse; PC-oil, oil-injected Prom1lacZ,DTA/+;CreERTM mouse; CreER-TM, tamoxifen-injected CreERTM mouse; Prom1-TM, tamoxifen-injected Prom1lacZ,DTA/+ mouse. (B) Upper panels show X-gal staining of frozen sections of tamoxifen-injected Prom1lacZ,DTA/+;CreERTM mouse brains. Lower four panels indicate PSA-NCAM immunostaining (red) in the ventricular zone and olfactory bulbs in tamoxifen-injected Prom1lacZ,DTA/+;CreERTM mice. Arrows show migrating neuroblasts in rostral migratory stream. All nuclei were counterstained with DAPI (blue). LV, lateral ventricle. Scale, 100 µm. (2.25 MB TIF) [file pone.0006869.s003.tif]

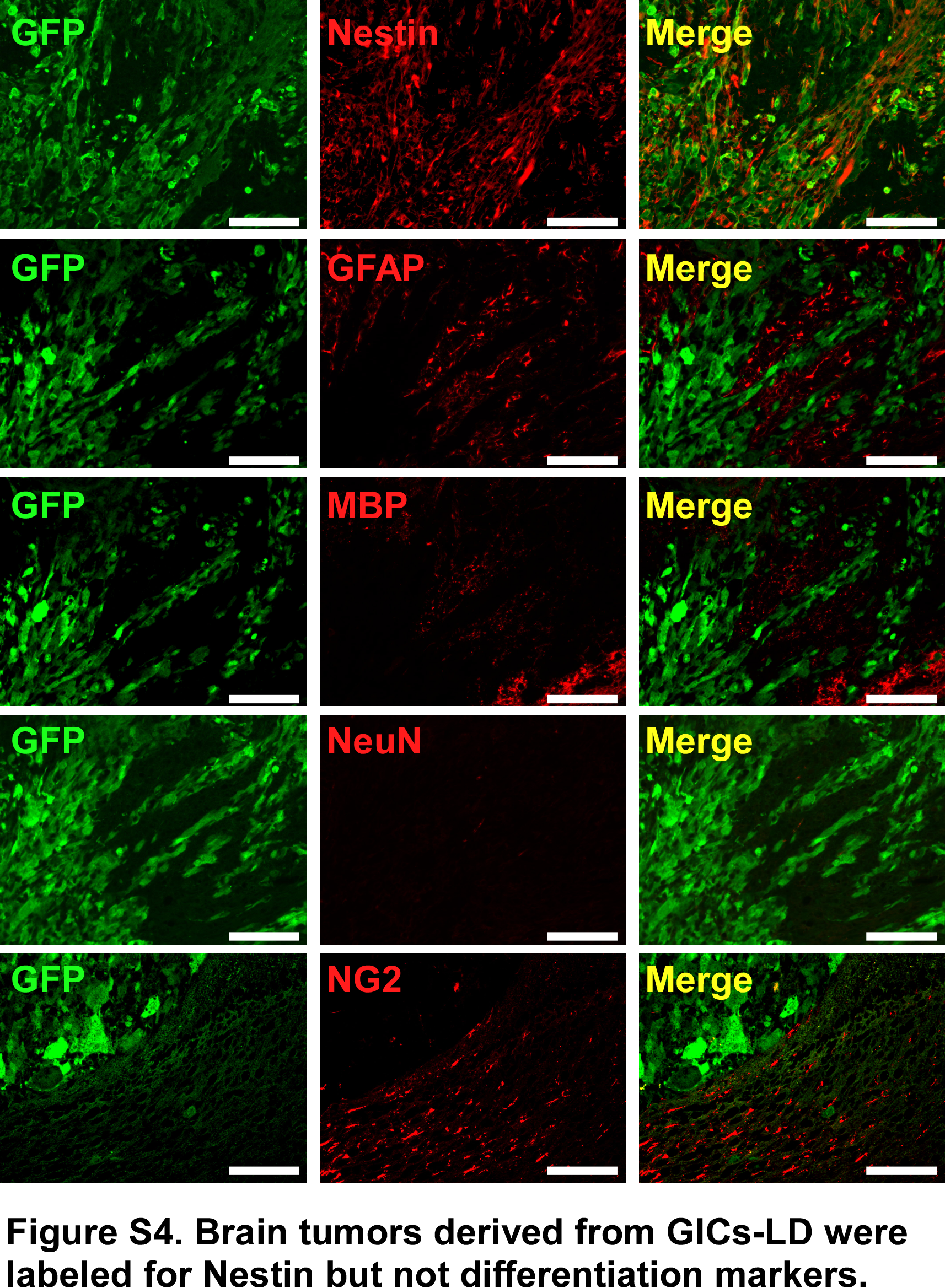

Supplement: Figure S4 — Brain tumors derived from GICs-LD were labeled for Nestin but not differentiation markers. Brain sections with tumors were immunolabeled for GFP (GICs, green) and either neural stem cell marker (Nestin, red) or differentiation markers, GFAP (astrocytes, red), MBP (oligodendrocytes, red), NeuN (mature neurons, red) and NG2 (oligodendrocytes precursor cells, red). Although most of GFP-positive cells were NG2-negative, very few double-positive cells were detected. Scale, 100 µm. (5.61 MB TIF) [file pone.0006869.s004.tif]

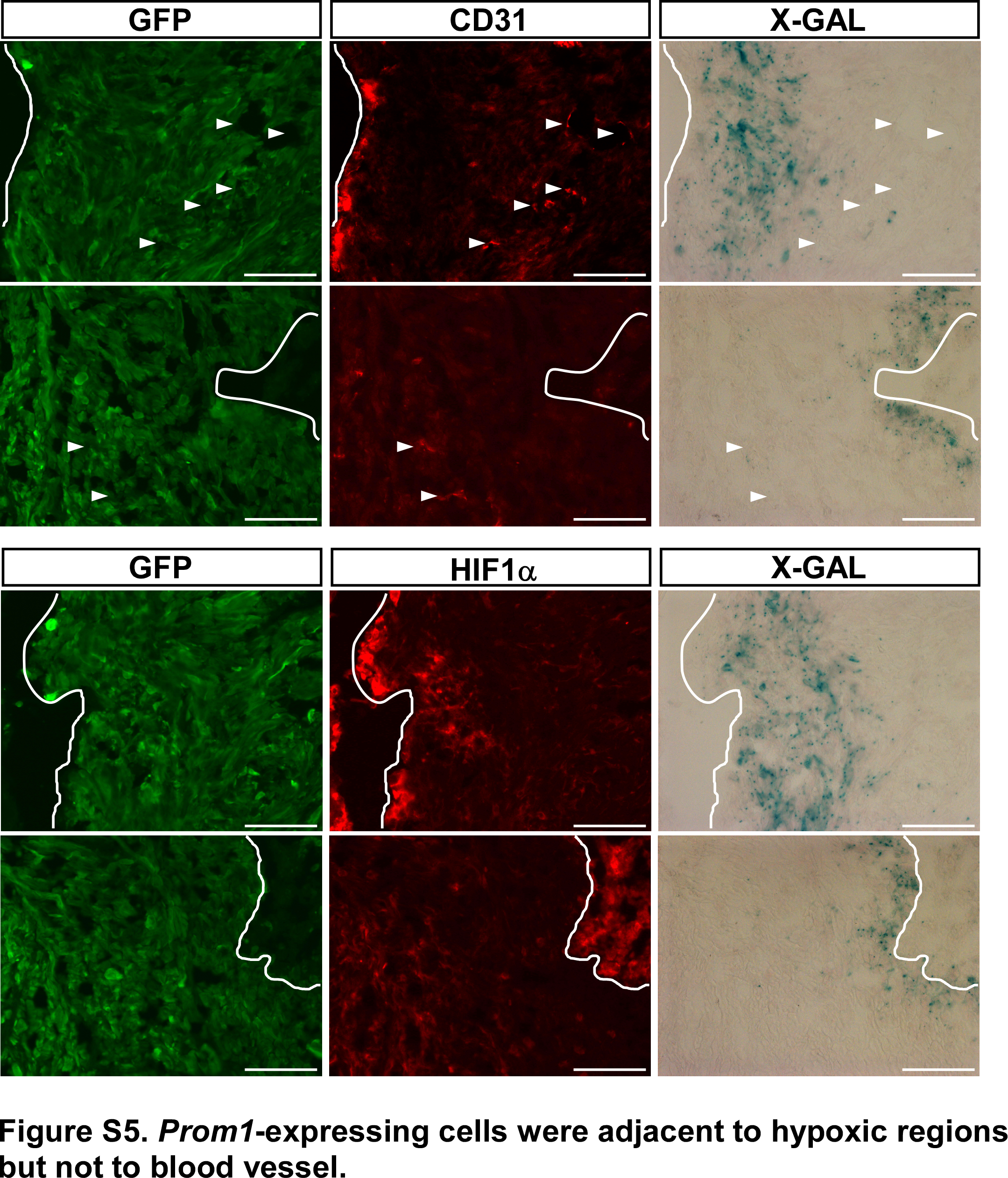

Supplement: Figure S5 — Prom1-expressing cells were adjacent to hypoxic regions but not to blood vessel. Frozen sections of brain tumors were stained with X-gal activity and then immunoelabeled for GFP (green) and either CD31 (marker for endothelrial cells, red) or HIF1α (marker for hypoxia, red). Arrow heads indicate CD31-positive cells. Scale, 100 µm. (9.60 MB TIF) [file pone.0006869.s005.tif]
